# Supplementary material for: Inverse problems for dynamic patterns in coupled oscillator networks: when larger networks are simpler
Source: Nat Commun. 2026 Feb 27;17:2075. doi: 10.1038/s41467-026-70016-y (PMC12949230; doi:10.1038/s41467-026-70016-y)
Supplement: Supplementary file 1 — Supplementary Information [file 41467_2026_70016_MOESM1_ESM.pdf]

Supplementary Information to the manuscript

"Inverse problems for dynamic patterns in coupled oscillator networks: When larger networks are simpler"

Oleh E. Omel'chenko[1,*]

[1] *Institute of Physics and Astronomy, University of Potsdam, Karl-Liebknecht-Str. 24/25,14476 Potsdam,Germany*

SUPPLEMENTARY NOTE 1: ON THE OPTIMAL NUMBER OF FOURIER MODES IN THE REPRESENTATION OF THE COUPLING FUNCTION $G(x)$

For a given set of local order parameters ζ_k and effective frequencies Ω_k, the L^2-accuracy of the reconstruction of the coupling function $G(x)$ increases almost monotonically with the number of Fourier modes M [see Eq. (14) in the manuscript], but only up to some threshold value M_{opt}, while for $M > M_{\text{opt}}$ the accuracy starts to deteriorate, see Supplementary Fig. 1 below. Remarkably, the value M_{opt} is not related to the Gibbs phenomenon, but to the finite accuracy of the statistical equilibrium relations. Since these relations are exact for an infinite number of oscillators N and an infinite averaging time T, we know that $M_{\text{opt}} \to \infty$ as $N, T \to \infty$. But a more specific functional dependence of M_{opt} on N and T is difficult, if not impossible, to obtain.

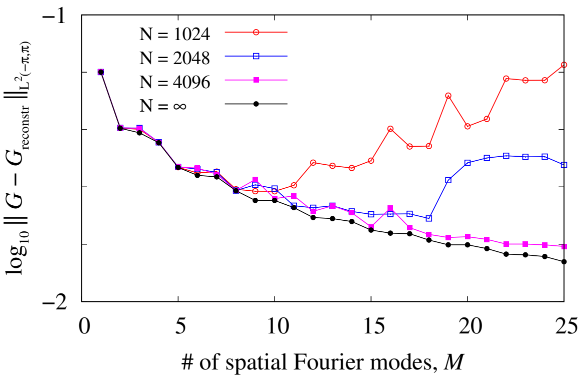

SUPPLEMENTARY FIG. 1. L^2-accuracy of the reconstructed coupling function $G_{\text{reconstr}}(x)$ relative to the original coupling function $G(x)$. The chimera state for the parameters from Fig. 2 in the manuscript was numerically simulated for system sizes $N = 1024$, 2048, and 4096, and the corresponding effective frequencies Ω_k and local order parameters ζ_k were calculated as time-averaged over $T = 2000$. The obtained data were used in our reconstruction algorithm with different M. In each case, the accuracy initially increases with M, but for the number of Fourier harmonics M greater than some M_{opt} starts to deteriorate. The black curve shows the maximum possible accuracy provided by a truncated Fourier series with all spatial modes $\cos(mx)$ where $m \leq M$.

* Corresponding author: omelchenko@uni-potsdam.de
