# Supplementary material for: Inverse problems for dynamic patterns in coupled oscillator networks: when larger networks are simpler
Source: Nat Commun. 2026 Feb 27;17:2075. doi: 10.1038/s41467-026-70016-y (PMC12949230; doi:10.1038/s41467-026-70016-y)
Supplement: Supplementary file 2 — Description of Additional Supplementary Files [file 41467_2026_70016_MOESM2_ESM.pdf]

Description of the Supplementary Data 1 file

attached to the manuscript "Inverse problems for dynamic patterns in coupled oscillator networks: When larger networks are simpler"

Oleh E. Omel'chenko[1,*]

[1] *Institute of Physics and Astronomy, University of Potsdam, Karl-Liebknecht-Str. 24/25,14476 Potsdam,Germany*

The file "SupplementaryData1.zip" contains the MatLab codes that generate the data for the figures in the manuscript.

1) The folder "Codes/Dynamics" contains a MatLab-script "Dynamics.m" and two auxiliary files. By running this script, one obtains the chimera state shown in Figure 2(a). In particular, the final state snapshot is saved in the file "FinalData.dat".

2) The folder "Codes/Simulation" contains a MatLab-script "Simulation.m" and two auxiliary files. Running this script calculates the effective frequencies and local order parameters for the chimera state shown in Figure 2. The results are saved in the file "Data.dat".

3) The folder "Codes/Reconstruction" contains a MatLab-script "Reconstruction.m" and five auxiliary files. By running this script, one obtains the data shown in Figure 4. Note that the "Data.dat" file containing the effective frequencies and local order parameters must be present in the working folder!

* Corresponding author: omelchenko@uni-potsdam.de
